# Influence of Structured Plasma-Based Composition on Functional, Textural and Sensory Characteristics of Emulsion-Type Sausages

**DOI:** 10.3390/foods15081336

**Published:** 2026-04-12

**Authors:** Amirzhan Kassenov, Assem Shulenova, Mukhtarbek Kakimov, Gulnara Kokayeva, Ayaulym Mustafayeva, Bauyrzhan Iskakov, Serik Tokayev, Maigul Mursalykova, Yelena Krasnopyorova, Diana Sviderskaya

**Affiliations:** 1 Institute of Engineering and Food Technology, S. Seifullin Kazakh Agro Technical University, Astana 010011, Kazakhstan; amirzhankassenov@yandex.kz (A.K.); muhtarbek@mail.ru (M.K.); g.kokaeva@kazatu.edu.kz (G.K.); ayaulym.mustafa7878@gmail.com (A.M.); baissemey@bk.ru (B.I.); tokaev_sd@mail.ru (S.T.); 2 Department of Technological Equipment, Shakarim University, Semey 071412, Kazakhstan; mursalykovamaigul@gmail.com; 3 Department of Engineering and Industrial Technologies, Innovative University of Eurasia, Pavlodar 140000, Kazakhstan; kef.171080@gmail.com; 4 Department of Architecture and Design, Toraigyrov University, Pavlodar 140000, Kazakhstan; sofilsev@rambler.ru

**Keywords:** structured composition, blood plasma proteins, cooked sausage, meat replacement, texture properties, amino acid profile, sensory quality

## Abstract

This study investigated the technological feasibility of using a pre-structured bovine blood plasma–flaxseed composition as a functional partial substitute for beef in emulsion-type sausages. Five formulations containing 0–30% replacement were evaluated to determine effects on structural, nutritional, and microbiological properties. Incorporation of the structured composition modified the functional balance of the protein system: water-holding capacity remained stable (*p* > 0.05), while fat-holding and emulsifying capacities improved at higher inclusion levels (*p* < 0.05), indicating enhanced interfacial stabilization of the fat phase. Progressive softening of texture was observed, with significant reductions in hardness and chewiness at 30% replacement (*p* < 0.05). Cooking loss increased at elevated substitution levels but remained within acceptable technological limits. During refrigerated storage, microbial counts remained below safety thresholds. A 15–25% replacement level provided the most balanced performance, maintaining sensory acceptability while improving lipid stabilization. The results demonstrate that structured plasma-based systems can function as effective protein–emulsion modifiers in meat formulations, supporting sustainable valorization of slaughter by-products.

## 1. Introduction

The intensification of meat processing generates substantial volumes of slaughter by-products, among which blood represents one of the most problematic streams [1,2,3]. Blood is characterized by high biochemical activity, susceptibility to microbiological contamination in cases of inadequate sanitary control, and a significant environmental burden during disposal. At the same time, it is a concentrated source of high-quality animal protein. The plasma fraction is of particular technological interest due to the solubility and functional properties of albumins, globulins, and fibrinogen [4,5]. In modern sustainable food production, such raw material streams are increasingly regarded as sources of high-value ingredients rather than waste, as emphasized in studies on meat industry sustainability and by-product valorization [6]. Blood plasma proteins exhibit pronounced water-binding capacity, emulsifying activity, and gel-forming ability. These properties make them promising structure-forming components in meat emulsion systems, including emulsion-type sausages, frankfurters, and pâtés [7,8,9]. Previous studies have systematized approaches to blood collection and processing and demonstrated that the functional properties of plasma proteins largely determine their technological applicability [10]. Applied research indicates that the incorporation of animal blood into sausage formulations affects physicochemical, textural, and sensory properties. Moderate inclusion levels can improve product quality and acceptability, whereas excessive amounts may negatively influence technological and sensory characteristics [11].

The broader implementation of blood plasma in meat products remains limited by several technological constraints. These include variability in composition and functionality depending on collection and processing conditions, the risk of off-flavor and off-odor formation, and the need to ensure microbiological safety and storage stability [12,13]. As a result, recent research has focused on targeted plasma structuring through fermentation with starter cultures, enzymatic modification, and combination with polysaccharides or hydrocolloids [14]. Biotechnological approaches allow control of acidity, water retention, and texture by reorganizing the protein network. They can also reduce sensory defects. This occurs due to fermentation metabolites and changes in volatile compounds [15,16].

Fermentation of blood plasma with lactic acid bacteria is widely studied as a structuring method. Acidification promotes protein aggregation and network formation, while starter culture enzymes can strengthen the gel structure [17,18]. It is important to control fermentation to avoid excessive acidity and undesirable flavor in emulsion-type sausages [19]. Fermented plasma can be used not as a separate product, but as a structured functional ingredient incorporated into the meat batter [20,21].

Another important research direction involves protein–polysaccharide networks and emulsion gels, which improve water and fat retention, emulsion stability, and texture in meat systems [22,23,24,25]. Combining proteins with plant hydrocolloids allows better control of elasticity and reduces syneresis. Flaxseed products, particularly flaxseed cake, are promising polysaccharide sources due to their high water-holding capacity and ability to stabilize emulsions, which can enhance juiciness and reduce thermal losses in meat products [26].

For emulsion-type sausages, it is essential to maintain a typical sensory profile while improving emulsion stability and texture under standard industrial conditions [27,28,29]. Although blood plasma can enhance technological performance, sensory risks require controlled formulation and substitution levels [30]. However, the combined use of fermented plasma and flax-derived ingredients as a pre-structured meat substitute in emulsion-type sausages remains insufficiently studied, particularly with respect to optimal replacement level and its effect on product quality. Although blood plasma and flax ingredients have been studied separately, their combined use as a fermented pre-structured meat substitute in emulsion-type sausages remains insufficiently investigated. Information is limited on the optimal replacement level and its effects on emulsion stability, texture, and overall product quality [31,32,33].

Accordingly, this study aimed to develop emulsion-type sausages with partial replacement of meat using a structured functional composition based on bovine blood plasma and to evaluate the effects of different incorporation levels on the chemical composition, physicochemical and technological properties, texture, sensory characteristics, amino acid and fatty acid profiles, and microbiological stability of the final product. The novelty of the study lies in the application of a structured plasma-based composition as a multifunctional meat substitute in cooked sausage formulations and in the integrated assessment of its influence on product quality. This approach is important for the field because it expands current knowledge on the use of animal by-products as functional food ingredients and supports the development of more sustainable meat technologies while maintaining technological performance and consumer acceptability.

## 2. Materials and Methods

### 2.1. Samples

Beef used for sausage production was obtained from the Altyn-Kazyk farm (Novomarkovka village, Ereymentau district, Akmola region, Kazakhstan). The cattle were pasture-fed on natural grasses. Premium-grade beef (protein 20.4 g/100 g, fat 2.5 g/100 g, ash 1.1 g/100 g) from the thigh of Akbas cattle was used in the formulations.

Fresh bovine blood was collected from Akbas bulls (under three years of age) at a slaughter facility. Cattle blood contains 17.3 g/100 g total protein, 80.9 g/100 g water. Immediately after collection, blood was transferred into vacuum-sealed sterile containers and transported under refrigerated conditions (2–6 °C). To prevent coagulation and ensure uniformity, partial hemolysis was induced by adding filtered water (5% of blood volume) at 25–35 °C.

Plasma was obtained under laboratory conditions by centrifugation of cooled blood at 3500 rpm for 10–15 min to ensure complete separation of erythrocytes. The supernatant plasma fraction was carefully collected and used for further experiments. The bovine plasma fraction contains 7.25 g/100 g protein substances.

Starter cultures were prepared from lyophilized concentrates of *Pediococcus pentosaceus* (Qingzhou, China; 1.0 × 10^10^–10^11^ CFU/g), *Bifidobacterium lactis* (BB12, Chr. Hansen, Nienburg, Germany), *Bifidobacterium longum* (BB46, Chr. Hansen, Nienburg, Germany), and *Staphylococcus xylosus* (MIC SALSA-1, Chr. Hansen, Hørsholm, Denmark). These cultures are commonly used in food fermentation and are characterized by acid-forming capacity, proteolytic activity, and the ability to enhance microbial stability and flavor development. All strains used in this study have a recognized safety status for food applications. Lactic acid bacteria and pediococci are included in the Qualified Presumption of Safety (QPS) list of the European Food Safety Authority, while *Bifidobacterium lactis BB12* and *Bifidobacterium longum BB46* are classified as Generally Recognized as Safe (GRAS) by the U.S. Food and Drug Administration. *Staphylococcus xylosus* is also widely accepted for use in fermented meat products and holds a favorable safety status for food applications.

Flaxseed cake was purchased from a local retail supplier in Astana, Kazakhstan. The chemical composition of flaxseed cake (per 100 g) was as follows: protein 26 g, fat 10 g, carbohydrates 12 g, dietary fiber 9.3 g, moisture 15 g, and ash 6.6 g. The energy value was 242 kcal. To enhance moisture retention and structural stability, flaxseed cake was incorporated as a hydrophilic plant component. Due to its high protein and dietary fiber content, flaxseed cake exhibits pronounced water-binding capacity, contributing to stabilization of the plasma protein matrix and formation of a structured protein–polysaccharide system.

### 2.2. Technology for Preparing a Structured Composition Based on Blood Plasma

**Figure 1:**
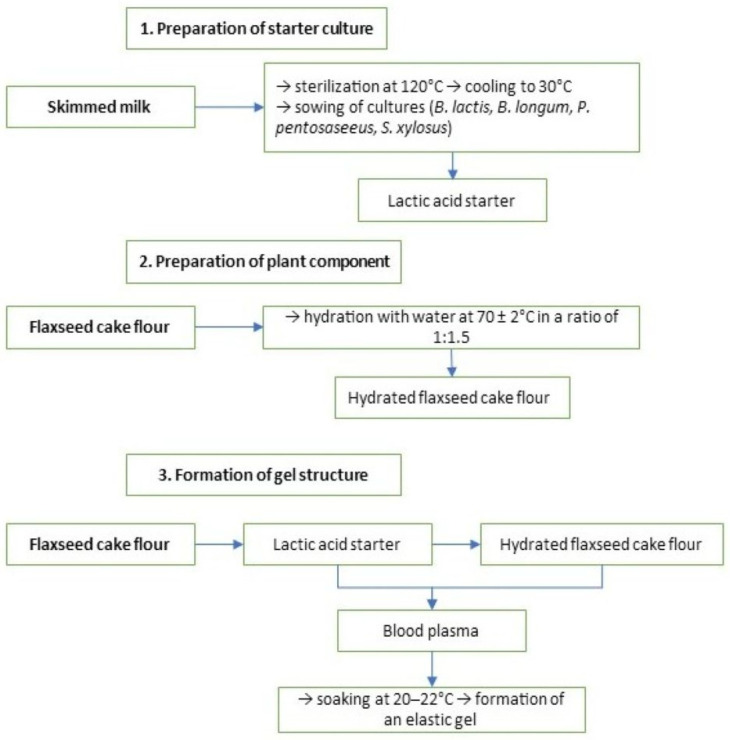
Technological scheme for the preparation of a structured composition based on blood plasma.

The technology for producing the structured composition incorporated into emulsion-type sausages is presented in Figure 1. The process was developed based on previously reported approaches to plasma structuring and protein–polysaccharide gel formation [34].

**Figure 2:**
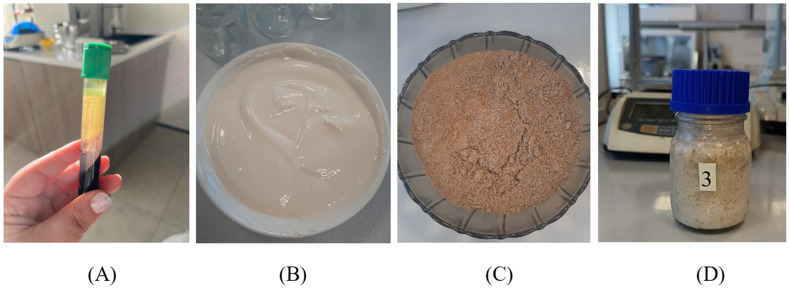
Components for preparing a composition based on bovine blood plasma: (**A**) Blood plasma obtained by centrifugation; (**B**) Inoculated milk; (**C**) Flaxseed meal; (**D**) Blood plasma-based composition.

The structured composition was obtained using bovine blood plasma, hydrated flaxseed cake flour, and a mixed starter culture consisting of *Bifidobacterium lactis*, *Bifidobacterium longum*, *Pediococcus pentosaceus*, and *Staphylococcus xylosus* (Figure 2). The technological scheme included three main stages: preparation of the starter culture, preparation of the plant component, and formation of the gel structure.

Skim milk was sterilized at 120 °C (approximately 1.2 atm) to ensure microbiological safety, cooled to 30 °C, and inoculated with lyophilized starter cultures, followed by incubation at 37 °C for 12–24 h to obtain an active lactic acid starter. Flaxseed cake flour was hydrated with water at 70 ± 2 °C in a ratio of 1:1.5 (flour:water), promoting swelling of polysaccharides and proteins and resulting in the formation of a viscous mass with high water-binding capacity. The prepared lactic acid starter was then added to blood plasma at the specified concentration (*w*/*w*), followed by incorporation of the hydrated flaxseed component. The mixture was subjected to mechanical stirring until a homogeneous system was obtained and subsequently maintained at 20–22 °C to ensure uniform distribution of microorganisms and stabilization of the protein–polysaccharide matrix prior to gel formation.

The structured blood plasma–flaxseed composition was characterized by the following proximate composition: moisture 75.5%, protein 16.7%, fat 1.3%, ash 1.8%, and carbohydrates 4.7%. The pH after fermentation was approximately 5.3. The composition demonstrated high techno-functional properties, including water-holding capacity (WHC) of 135% and fat-holding capacity (FHC) of 33%, indicating strong moisture retention and emulsion stability. The structured system formed a stable protein–polysaccharide gel with reduced syneresis (<10%) due to interactions between plasma proteins and flaxseed polysaccharides [34].

### 2.3. Preparation of Sausage Samples

The formulation was developed according to standard industrial processing schemes for cooked sausages. The structured plasma-based composition was incorporated into the minced meat system during comminution in a cutter. To ensure homogeneous distribution, the composition was added gradually during the liquid phase stage and mixed under continuous high-speed comminution (5000–6000 rpm) until a uniform emulsion was obtained. After stuffing into casings, the sausage batons were held at 0–4 °C for 2 h to allow stabilization of the protein–polysaccharide matrix and interaction between muscle proteins and the structured composition. Thermal treatment was carried out in a climate chamber under a stepwise regime: initial drying at 50–55 °C for 20–30 min, followed by heating at 75–80 °C until the core temperature reached 72 °C. After thermal processing and cooling, the sausages were stored under refrigerated conditions at 4 ± 1 °C for 14 days. Samples were analyzed at defined time intervals (0, 7, and 14 days) to evaluate changes in microbiological properties during storage.

To determine the optimal inclusion level, the structured composition was partially replaced with the main meat raw material at different concentrations. Five variants were produced: one control sample and four experimental samples containing 15%, 20%, 25%, and 30% of the structured composition, respectively, with a proportional reduction in beef content (Table 1).

The control sample was prepared in accordance with GOST 23670–2019 [35] and contained beef, raw beef fat, egg mélange, salt, sugar, spices, sodium nitrite, and other standard curing ingredients.

### 2.4. Determination of Chemical Composition

The chemical composition of the samples was determined according to international standard methods. Moisture content was measured in accordance with ISO 1442 [36] by drying samples in a drying oven at 105 °C to constant mass, followed by calculation of mass loss.

Protein content was determined using the Kjeldahl method in accordance with ISO 937 [37]. The method included mineralization of organic matter, distillation of released ammonia, and calculation of nitrogen content, which was converted to protein using the appropriate conversion factor.

Total ash content was determined according to ISO 936 [38] by incinerating the samples in a muffle furnace at 550 ± 25 °C until complete combustion of organic matter. The remaining mineral residue was weighed and expressed as total ash content.

Fat content was determined using the Soxhlet extraction method in accordance with standard procedures for meat and meat products.

### 2.5. Determination of Carbohydrate Content

The mass fraction of carbohydrates was determined by calculation as the difference between 100% and the sum of the main proximate components. Carbohydrate content (*X*, %) was calculated using Equation (1):

*X* = 100 – *A* – *B* – *C* − *D* (1)

where *A* is the mass fraction of moisture (%);

*B* is the mass fraction of protein (%);

*C* is the mass fraction of fat (%);

*D* is the mass fraction of ash (%).

The obtained value represents the total carbohydrate content expressed as a percentage of the product mass.

### 2.6. Determination of pH

The pH value was determined by the potentiometric method using a glass electrode in accordance with standard procedures [39]. The method is based on measuring the electromotive force generated between a reference electrode with a known potential and a glass electrode sensitive to hydrogen ion activity in the sample. Before analysis, the pH meter (pH-tester 340, Infraspak Analit, Novosibirsk, Russia) was calibrated using standard buffer solutions. The pH value was measured before and after a 2 h holding period at 0–4 °C prior to thermal treatment.

### 2.7. Determination of Water-Holding and Fat-Holding Capacity

Water-holding capacity (WHC) and fat-holding capacity (FHC) were determined according to the method described in [40].

### 2.8. Determination of Emulsifying Capacity

Emulsifying capacity (EC) was determined using a rotor–stator homogenization method. A 7 g sample was dispersed in 100 mL of distilled water and homogenized using an Ultra-Turrax homogenizer (IKA, Staufen, Germany) at 10,000 rpm for 60 s. Subsequently, 100 mL of refined sunflower oil was gradually added, and homogenization was continued at 12,000 rpm for 5 min to obtain a uniform oil-in-water emulsion. All procedures were performed at (20 ± 1) °C. The emulsion was transferred into four 50 mL centrifuge tubes and centrifuged at 3000× *g* for 10 min. After centrifugation, the volume of the emulsified oil layer (*V*_1_) was measured.

Emulsifying capacity (EC, %) was calculated according to Equation (2):

(2) $$EC=\frac{V_{1}}{V}\cdot 100$$

where

*V*_1_—volume of emulsified oil (mL);

*V*—total volume of added oil (mL).

### 2.9. Cooking Loss Determination

Cooking loss was determined by weighing the sausage samples before and after thermal treatment. Raw sausage batons were weighed prior to cooking, and after heat treatment and cooling to room temperature, the samples were reweighed after removing surface moisture. Cooking loss (*CL*, %) was calculated as the percentage difference between the initial and final weights using Equation (3):

(3) $$CL=\frac{W_{1}-W_{2}}{W_{1}}\cdot 100$$

where

*W*_1_—the weight of the sample before cooking;

*W*_2_—the weight after cooking.

### 2.10. Determination of Shear Stress and Cutting Work

Shear stress (*τ*) and cutting work (*A_cut_*) were determined using an Instron 6800 Series universal testing machine.

Cylindrical samples were prepared using a hollow punch with a diameter of 24.6 × 10^−3^ m and cut into discs approximately 10 × 10^−3^ m in height. Four discs were placed side by side at the bottom of a cubic shear chamber. The chamber was closed with a lid equipped with guiding grooves and positioned under a ten-bladed knife so that the blades aligned with the grooves. The chamber was then fixed in place, and the traverse movement was initiated.

The maximum cutting force (*P_max_*) was obtained from the force–deformation curve recorded during the test. Shear stress (τ, Pa) was calculated using Equation (4):

(4) $$\tau =\frac{P_{max}\cdot d}{\pi R^{2}\cdot h_{avg}\cdot n}$$

where *P_max_* is the maximum force recorded during cutting (N);

*d* is the width of the vertical groove (m);

*R* is the radius of the sample (m);

*h_avg_* is the average sample height (m);

*n* is the number of discs placed in the chamber.

Because samples with similar shear stress values may differ in perceived firmness during mastication, cutting work (*A_cut_*, J/m^2^) was additionally determined as an indicator of texture. Cutting work was calculated from the area under the force–deformation curve using Equation (5):

(5) $$A_{cut}=\frac{S\cdot V_{\tau p}\cdot d}{V_{l}\cdot \pi R^{2}\cdot h_{avg}\cdot n}$$

where

*S* is the area under the force–deformation curve (N·m), bounded by the moment when the ten-bladed knife passed completely through the sample (at 52 × 10^−3^ m from the intersection of the curve with the *p* = 0 axis);

*V_τp_* is the traverse speed (m/s);

*V_l_* is the chart recording speed (m/s).

### 2.11. Texture Profile Analysis

Texture profile analysis (TPA) of cooked sausage samples was performed using a TMS-Pro texture analyzer (Food Technology Corporation, Sterling, VA, USA) equipped with a 250 N load cell. Prior to testing, samples were equilibrated at (20 ± 1) °C for 1 h to ensure structural stabilization and temperature uniformity. Cylindrical specimens (25 mm diameter, 15 mm height) were excised from the central portion of each sausage to avoid edge effects and structural heterogeneity. Samples were subjected to a double-compression test simulating mastication. The compression was carried out to 50% of the original height at a constant crosshead speed (specify speed, e.g., 1.0 mm/s if applicable) with a flat cylindrical probe. A 5 s interval between the first and second compression cycles was maintained.

### 2.12. Determination of Amino Acid Composition

The amino acid composition of sausage samples was determined by capillary electrophoresis (CE) using a Kapel-105M system (Lumex, Saint Petersburg, Russia). Samples were subjected to acid hydrolysis in 6 M hydrochloric acid at 110 °C for 24 h to convert bound amino acids into free forms. After hydrolysis, the solutions were filtered and evaporated to remove excess hydrochloric acid. The residues were dissolved in an appropriate buffer solution for further derivatization. For the determination of proteinogenic amino acids (except tryptophan), phenylisothiocyanate (PITC) derivatives were obtained prior to separation. Separation was performed in a fused-silica capillary (total length 75 cm, internal diameter 50 μm) using a phosphate background electrolyte containing β-cyclodextrin. The applied voltage was +25 kV, capillary temperature was maintained at 30 °C, and UV detection was carried out at 254 nm. Tryptophan was determined separately without derivatization under borate buffer conditions with UV detection at 219 nm. Quantification was performed using calibration with standard amino acid solutions. Results were expressed as g per 100 g of product (or % of total protein).

### 2.13. Determination of Fatty Acid Composition

The fatty acid composition of the sausage samples was determined by gas chromatography with flame ionization detection (GC–FID). The analysis was performed using a Kristallux-4000M gas chromatograph equipped with a flame ionization detector (Meta-Chrom, Yoshkar-Ola, Russia). Chromatographic data acquisition and processing were carried out using NetChrom v2.1 software. Before analysis, the lipid fraction was extracted from the samples. The isolated fatty acids were converted into fatty acid methyl esters (FAME) using standard derivatization procedures. The obtained methyl esters were injected into the chromatographic system. Separation was carried out on a capillary column under a programmed temperature regime. Fatty acids were detected using the flame ionization detector. Identification was performed by comparing retention times with those of standard FAME mixtures. Quantification was based on normalization of chromatographic peak areas and expressed as a percentage of total identified fatty acids.

### 2.14. Determination of Microbiological Safety Indicators

Microbiological safety of the cooked sausage samples was evaluated in accordance with ISO 7218:2024, which establishes general requirements for microbiological examination of food products [41]. The total viable count (TVC) of mesophilic aerobic and facultative anaerobic microorganisms was determined by plate count at 30 °C. Lactic acid bacteria (LAB) were enumerated on MRS agar with incubation at 30 °C. Sanitary-indicative microorganisms were assessed by enumeration of *Enterobacteriaceae* and coliform bacteria using standard culture-based methods. Coagulase-positive staphylococci were determined with confirmation of coagulase activity. Yeasts and molds were also enumerated using selective media. The presence of pathogenic microorganisms, including *Salmonella* spp. and *Listeria monocytogenes*, was assessed using standard detection procedures. Quantitative results were expressed as colony-forming units per gram (CFU/g), while pathogens were reported as “not detected” in accordance with applicable regulatory requirements.

### 2.15. Determination of Organoleptic Indicators

Organoleptic properties of the sausage samples were evaluated in accordance with [42]. Sensory evaluation was conducted by a panel of 15 trained experts under controlled laboratory conditions, in a room free from extraneous odors and under neutral lighting. The samples were assessed according to the following attributes: appearance, color, odor, taste, and consistency. Evaluation was performed using a five-point scale, where 5 points indicated full compliance with regulatory and quality requirements, and 1 point indicated unsatisfactory quality. Since sensory scores represent ordinal data and may not follow a normal distribution, non-parametric statistical methods were applied. Differences among five sample groups (one control and four experimental variants) were first analyzed using the Kruskal–Wallis test. When significant differences were detected, pairwise comparisons with the control sample were performed using the Mann–Whitney test with Holm correction for multiple comparisons. The experimental protocol was approved by Local Ethical Commission of the Institutional Review Board of S. Seifullin Kazakh Agro Technical University, Astana, Kazakhstan, with the approval number PLEC VND 03.3017-21 and approval date 21 September 2023.

### 2.16. Statistical Analysis

Statistical analysis was performed to evaluate differences between the control formulation and experimental sausages containing 15–30% structured composition based on blood plasma. The experiment was conducted with five formulation variants (control and four experimental samples). For each variant, eight independent sausage units (300–350 g each) were produced (*n* = 8). Each sausage unit was considered an independent experimental replicate. For each replicate, analytical determinations were carried out in triplicate (*n* = 3), and the mean value of the three measurements was used for statistical analysis. Data were analyzed using two-way analysis of variance (ANOVA) to evaluate the effects of formulation (control, Sample 1–4), storage time (0, 7, and 14 days), and their interaction on the studied parameters. When significant differences were detected (*p* < 0.05), mean comparisons were performed using Tukey’s HSD test.

Results are presented as mean ± standard deviation. For sensory evaluation, non-parametric analysis was applied. The Kruskal–Wallis test was used to assess overall differences among samples, followed by pairwise comparisons where appropriate. All statistical analyses were performed using SPSS v. 28 (IBM Corp., Armonk, NY, USA). Differences were considered statistically significant at *p* < 0.05.

## 3. Results

### 3.1. Studying the Chemical Composition of Sausages

The proximate composition of sausage variants with partial replacement of beef by a structured plasma-based composition is presented in Table 2. Moisture content increased significantly with increasing levels of the structured composition (*p* < 0.05), whereas protein and fat contents showed a gradual decrease (*p* < 0.05). Carbohydrate content remained relatively stable across all formulations, with no statistically significant differences observed (*p* > 0.05). In contrast, ash content showed a slight but significant increase with increasing inclusion levels of the structured composition (*p* < 0.05). Overall, incorporation of the structured composition resulted in higher moisture and ash contents, accompanied by reductions in protein and fat fractions.

### 3.2. Studying the pH Value of Sausage Variants

**Figure 3:**
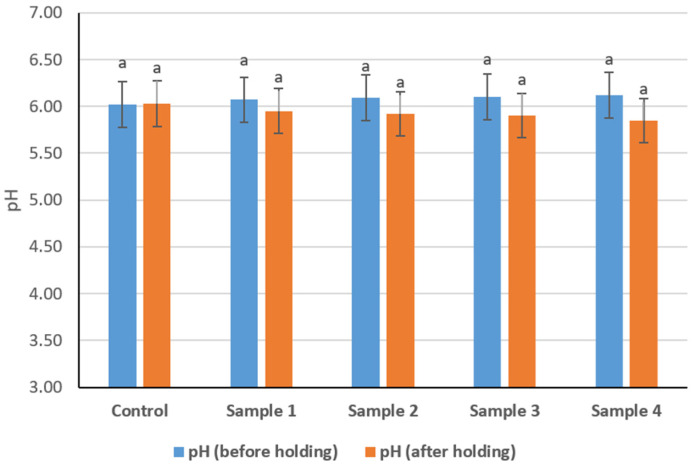
pH values of cooked sausage formulations before and after 2 h holding. (Similar lowercase letters above the bars indicate no statistically significant differences between samples (*p* > 0.05)).

The pH values of sausage variants measured before and after a 2 h holding period prior to thermal treatment are presented in Figure 3. Before holding, the pH values ranged from 6.02 in the control sample to 6.12 in Sample 4. A slight increase in pH was observed with increasing levels of the structured plasma-based composition. However, statistical analysis did not reveal significant differences among the samples (*p* > 0.05). After 2 h of holding, pH values decreased slightly in all formulations. The pH values after holding ranged from approximately 5.85 to 6.00, depending on the sample. Despite this decrease, no statistically significant differences were detected either between the formulations or between the measurements taken before and after holding (*p* > 0.05). The pH values of all sausage variants remained within a narrow range throughout the holding period.

### 3.3. Studying the Water-Holding and Fat-Holding Capacity of Sausage Variants

**Figure 4:**
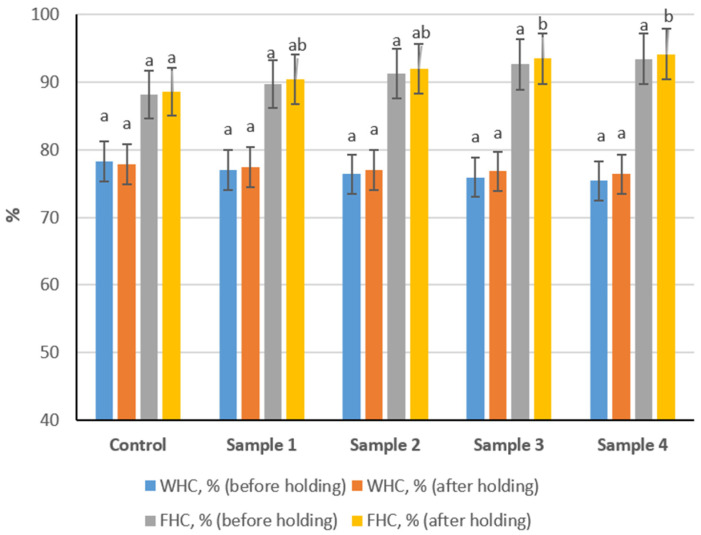
Water-holding and fat-holding capacity of cooked sausage formulations before and after 2 h holding. (Different lowercase letters above the bars indicate statistically significant differences between samples (*p* < 0.05)).

The water-holding capacity (WHC) and fat-holding capacity (FHC) of sausage variants measured before and after a 2 h holding period are presented in Figure 4. WHC values remained within a narrow range (approximately 75–79%), whereas FHC values ranged from about 88% to 95%. WHC showed a slight decreasing trend with increasing levels of the structured composition. However, no statistically significant differences were observed either before or after the holding period (*p* > 0.05). The 2 h holding step did not substantially affect WHC values across the samples. In contrast, FHC exhibited an increasing trend with higher inclusion levels of the structured composition. While differences before holding were not statistically significant (*p* > 0.05), after the holding period Samples 3 and 4 demonstrated significantly higher FHC compared to the control (*p* < 0.05).

### 3.4. Studying the Cooking Loss of Sausage Variants

**Figure 5:**
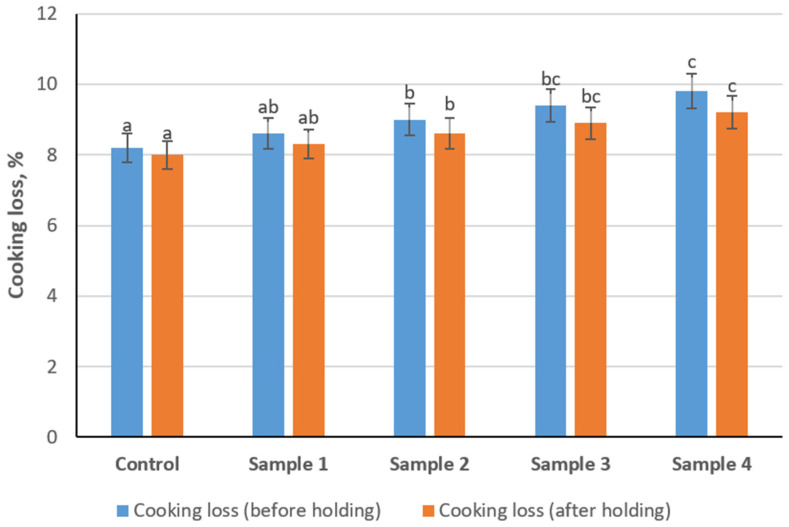
Effect of structured plasma-based composition and pre-heat holding on cooking loss of emulsion-type sausages. Different lowercase letters (a–c) above the bars indicate statistically significant differences between samples within the same measurement condition (*p* < 0.05).

Cooking loss of sausage variants measured before and after a 2 h holding period is presented in Figure 5. Cooking loss values ranged approximately from 8.0% to 9.8% across all samples. Before holding, cooking loss showed an increasing trend with increasing levels of the structured composition, with Samples 3 and 4 demonstrating significantly higher values compared to the control (*p* < 0.05). After the 2 h holding period, cooking loss decreased slightly in all formulations. However, Samples 3 and 4 remained significantly higher than the control (*p* < 0.05). Overall, cooking loss increased with increasing levels of the structured composition, while the holding step contributed to a slight reduction in cooking losses.

### 3.5. Studying the Emulsifying Capacity of Sausage Variants

**Figure 6:**
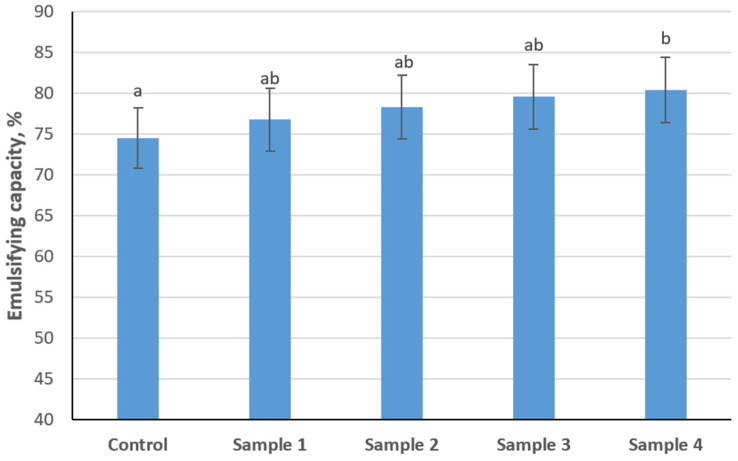
Effect of structured plasma-based composition level on emulsifying capacity of emulsion-type sausages. (Different letters indicate statistically significant differences (*p* < 0.05)).

The emulsifying capacity of sausage variants with different levels of structured plasma-based composition is presented in Figure 6. The control sample showed an emulsifying capacity of 74.5%. As the structured composition increased, the emulsifying capacity gradually increased, reaching 80.4% in Sample 4. However, only Sample 4 demonstrated a statistically significant increase in emulsifying capacity compared with the control sample (*p* < 0.05). Overall, an increasing trend in emulsifying capacity was observed with increasing replacement level.

### 3.6. Studying the Shear Stress and Cutting Work of Sausage Variants

The mechanical properties of emulsion-type sausages, expressed as shear stress and cutting work, are presented in Table 3. Shear stress decreased significantly with increasing levels of the structured plasma-based composition (*p* < 0.05). All experimental samples showed lower values compared to the control, with Sample 4 demonstrating the lowest shear stress. Differences between Samples 3 and 2 were not statistically significant (*p* > 0.05), while Sample 4 differed significantly from Samples 1 and 2 (*p* < 0.05). A similar pattern was observed for cutting work. All experimental variants exhibited significantly lower values compared to the control (*p* < 0.05), with the lowest values recorded in Sample 4. No significant difference was found between Samples 2 and 3 (*p* > 0.05), whereas Sample 4 differed significantly from Samples 1 and 2 (*p* < 0.05). Overall, both shear stress and cutting work decreased with increasing levels of the structured composition.

### 3.7. Texture Profile Analysis of Sausage Variants

Texture profile analysis (TPA) parameters of emulsion-type sausages with different levels of structured plasma-based composition are presented in Table 4. Hardness decreased progressively with increasing levels of the structured composition, with Samples 2–4 showing significantly lower values compared to the control (*p* < 0.05). A similar trend was observed for chewiness and gumminess, which decreased with increasing inclusion levels of the structured composition. Significant reductions in chewiness were observed in Samples 3 and 4 (*p* < 0.05), while gumminess also decreased significantly compared to the control (*p* < 0.05). Cohesiveness and springiness showed slight but statistically significant decreases at higher replacement levels (Samples 3 and 4) (*p* < 0.05). In contrast, adhesiveness did not differ significantly among the samples (*p* > 0.05). Increasing levels of the structured composition were associated with gradual softening of the product structure, as reflected by reductions in hardness, gumminess, and chewiness.

### 3.8. Studying the Amino Acid Composition of Sausage Variants

The essential amino acid composition of emulsion-type sausages with partial replacement of beef by a structured plasma-based composition is presented in Table 5. The total amount of amino acids increased gradually with increasing levels of the structured composition, rising from 37.13 in the control sample to 38.08 in Sample 4. Although a gradual increase in the total amino acid content was observed with increasing levels of the structured plasma-based composition, the differences between samples were not statistically significant (*p* > 0.05).

Among individual amino acids, valine increased from 4.12 in the control sample to 4.38 in Sample 4, with significant differences observed between the control and higher replacement levels (*p* < 0.05). Threonine also increased from 3.86 in the control to 4.20 in Sample 4 (*p* < 0.05). Phenylalanine + tyrosine showed a gradual increase from 6.37 in the control sample to 6.75 in Sample 4. However, no clear statistical separation among all variants was observed (*p* > 0.05). Leucine and lysine remained relatively stable across all formulations, showing no statistically significant differences among samples (*p* > 0.05). Isoleucine and tryptophan also did not differ significantly between variants (*p* > 0.05). Histidine showed a slight decrease from 2.80 in the control sample to 2.63 in Sample 4, with significant differences between the control and the highest replacement level (*p* < 0.05). Sulfur-containing amino acids (methionine + cysteine) decreased progressively with increasing levels of the structured composition, from 2.40 in the control sample to 2.14 in Sample 4. Statistical analysis showed significant differences between the control and higher replacement levels (*p* < 0.05).

### 3.9. Studying the Fatty Acid Composition of Sausage Variants

The fatty acid composition of emulsion-type sausages with partial replacement of beef by the structured blood plasma-based composition is presented in Table 6. Total fatty acid content increased significantly with increasing levels of the structured composition (*p* < 0.05). Among saturated fatty acids, short- and medium-chain fatty acids (C10:0, C12:0, and C14:0) showed a decreasing trend with increasing replacement level (*p* < 0.05), while palmitic acid (C16:0) remained relatively stable (*p* > 0.05). In contrast, stearic acid (C18:0) increased significantly with increasing levels of the structured composition (*p* < 0.05). Among unsaturated fatty acids, palmitoleic acid (C16:1) and linoleic acid (C18:2 n-6) did not show significant changes among the samples (*p* > 0.05). Minor but statistically significant variations were observed for α-linolenic acid (C18:3) and arachidonic acid (C20:4 n-6), although these changes were not pronounced.

Based on fatty acid grouping, saturated fatty acids (SFA) increased significantly in Samples 2–4 compared to the control (*p* < 0.05). Monounsaturated fatty acids (MUFA) showed limited variation, with only Sample 4 differing significantly from Sample 1 (*p* < 0.05). Polyunsaturated fatty acids (PUFA), as well as total ω-6 and ω-3 fatty acids, remained relatively stable across all formulations, with no statistically significant differences observed (*p* > 0.05).

### 3.10. Sensory Evaluation of Sausage Variants

**Figure 7:**
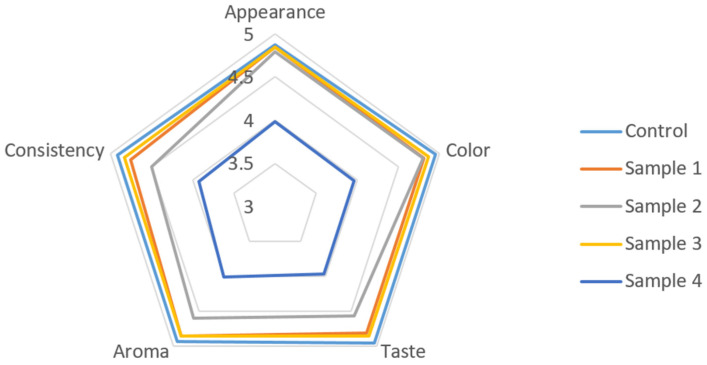
Sensory profile comparison of cooked sausage formulations.

**Figure 8:**
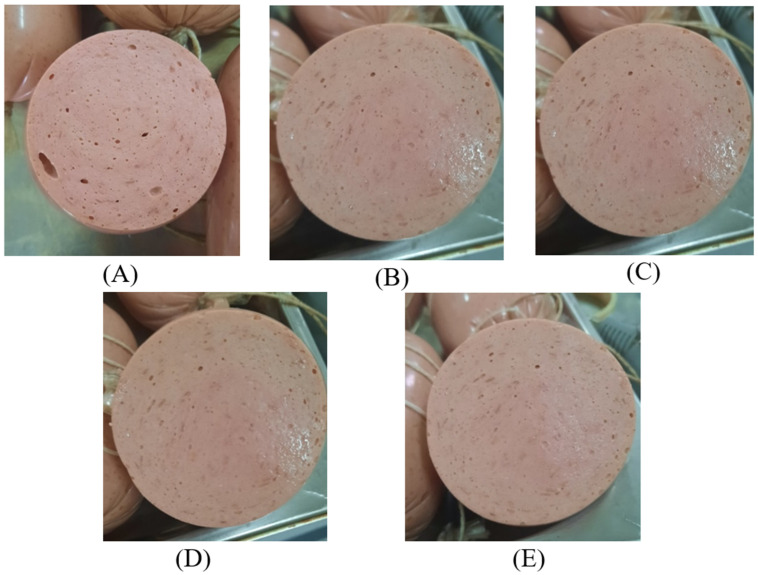
Cross-sectional appearance of cooked sausage samples: (**A**)—Control; (**B**)—Sample 1 (15% replacement); (**C**)—Sample 2 (20% replacement); (**D**)—Sample 3 (25% replacement); (**E**)—Sample 4 (30% replacement).

Sensory evaluation of sausage variants with partial replacement of beef by the structured plasma-based composition is presented in Figure 7. The evaluated attributes included appearance (Figure 8), color, taste, aroma, and consistency. The control sample received high scores for all attributes, ranging from 4.87 to 4.95 points. Samples 1 and 3 showed similar values across most sensory parameters and did not differ significantly from the control sample (*p* > 0.05).

Sample 2 demonstrated slightly lower scores for several attributes. Appearance and color scores were 4.79 and 4.81, respectively, while taste and aroma scores were 4.56 and 4.60. Some of these differences were statistically significant compared with the control (*p* < 0.05). Sample 4 showed the lowest sensory scores among all formulations. Appearance, color, and taste values were approximately 3.98, 3.96, and 3.96, respectively. Aroma and consistency scores were also lower than in other samples. These values were significantly lower than those of the control and other experimental variants (*p* < 0.05).

The overall sensory score was highest in the control sample (24.62 points). Samples 1 and 3 showed similar overall scores (24.08 and 24.25 points, respectively) and did not differ significantly from the control (*p* > 0.05). Sample 2 showed a slightly lower overall score (23.25 points), while Sample 4 demonstrated the lowest value (19.84 points) and differed significantly from the other samples (*p* < 0.05).

### 3.11. Microbiological Safety Indicators of Sausage Variants

**Figure 9:**
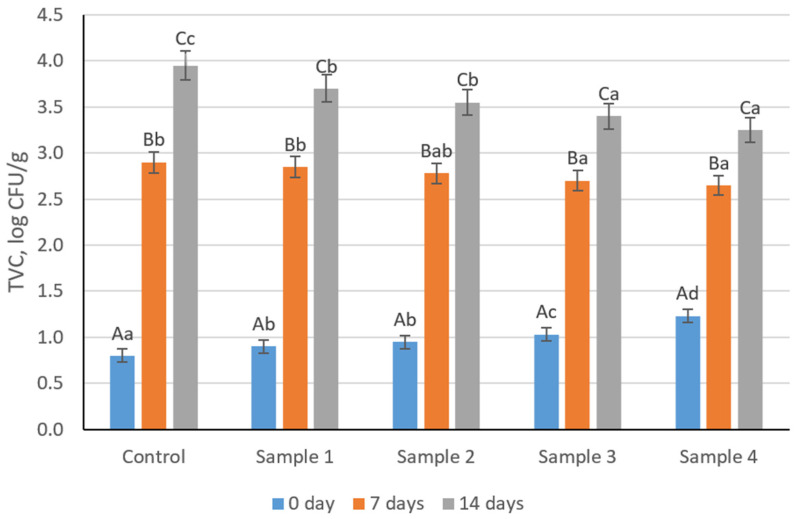
Changes in total viable count of cooked sausage samples during refrigerated storage. Uppercase letters indicate statistically significant differences within each sample across storage time, while lowercase letters indicate statistically significant differences between samples at the same storage time (*p* < 0.05).

**Figure 10:**
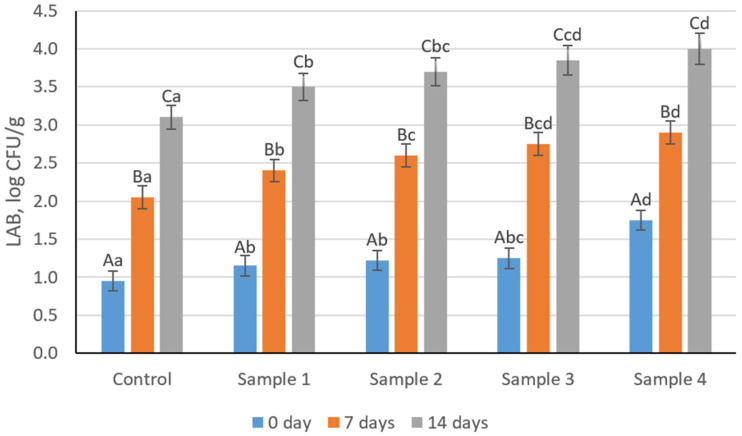
Changes in lactic acid bacteria of cooked sausage samples during refrigerated storage. Uppercase letters indicate statistically significant differences within each sample across storage time, while lowercase letters indicate statistically significant differences between samples at the same storage time (*p* < 0.05).

Microbiological indicators of sausage variants during refrigerated storage (4 ± 1 °C) are presented in Figure 9 and Figure 10 and Table S1 (Supplementary Materials). Total viable counts (TVC) increased significantly during storage in all samples (*p* < 0.05). The increase followed a typical time-dependent pattern, reflecting gradual microbial growth under refrigerated conditions. At the same time, samples containing the structured plasma-based composition tended to exhibit slightly lower TVC values at later storage stages compared to the control, suggesting a potential stabilizing effect of the modified system. A similar trend was observed for lactic acid bacteria (LAB), with a progressive increase during storage (*p* < 0.05). The increase in LAB was more pronounced in samples with the structured composition, which may be associated with the presence of residual starter culture activity and the availability of nutrients supporting bacterial growth. This indicates that the structured system may create a more favorable microenvironment for lactic acid bacteria development. Despite these changes, all microbial counts remained within acceptable limits for cooked sausage products throughout storage. Enterobacteriaceae remained below the detection limit, and no pathogenic or spoilage microorganisms were detected in any samples, confirming the microbiological safety and hygienic stability of the products.

## 4. Discussion

The results of this study show that partial replacement of beef with a structured composition based on bovine blood plasma affects the chemical composition, functional properties, texture, nutritional profile, sensory quality, and microbiological stability of emulsion-type sausages. The changes observed in the sausage system depended on the level of replacement. Moderate inclusion of the structured composition caused controlled modification of the protein–water–fat matrix, whereas excessive replacement weakened the heat-induced protein structure and reduced product quality. Thus, the effects observed in this study should be interpreted as structural reorganization of the sausage matrix rather than simple substitution of ingredients.

The observed increase in moisture content with increasing addition of the structured composition is associated with the introduction of additional water bound within the plasma–flaxseed system, which contains hydrophilic proteins and polysaccharides. These ingredients can promote the formation of a hydrated gel-like network capable of retaining immobilized water during heating [43]. At the same time, the gradual decrease in protein content reflects the replacement of myofibrillar muscle proteins by plasma-derived and plant-based components. Importantly, this decrease should not be interpreted as a direct loss of technological functionality, because plasma proteins also possess emulsifying and gel-forming properties that support structural stability of comminuted meat systems [44]. The reduction in fat content is consistent with the lower lipid contribution of the structured composition compared with beef raw material, while the relatively stable carbohydrate fraction indicates that the main compositional redistribution occurred among water, protein, and fat. The moderate increase in ash content most likely reflects the mineral contribution of blood plasma and flaxseed ingredients.

The pH behavior of the experimental sausages also supports the interpretation that the structured composition altered the physicochemical environment of the meat batter without causing destabilization. The slight upward tendency in pH before holding can be explained by the relatively alkaline character of bovine blood plasma [45]. Even small pH shifts may influence protein functionality by increasing electrostatic repulsion between myofibrillar proteins, promoting swelling, and improving hydration of the matrix [46]. The slight decrease after holding, although not statistically significant, may reflect redistribution of ions and progressive hydration of protein fractions before thermal processing. Thus, while pH changes were small, they remain technologically relevant because they may influence water retention and subsequent gel formation.

This interpretation is consistent with the functional data, as water-holding capacity showed only minor, statistically insignificant changes among samples (*p* > 0.05), indicating that the structured plasma-based composition did not significantly modify the intrinsic moisture retention properties of the meat matrix. This stability may result from compensation between two opposing mechanisms: on the one hand, partial dilution of salt-soluble myofibrillar proteins may reduce the ability of the meat system to immobilize water; on the other hand, plasma proteins and hydrated flax components can bind water and support retention within the matrix [47]. In contrast, fat-holding capacity and emulsifying capacity improved with increasing replacement level. These effects are consistent with the known interfacial activity of plasma proteins, especially albumins and globulins, which adsorb at the oil–water interface and reduce interfacial tension [48]. In addition, the plant-derived component likely increases the viscosity of the continuous phase, limiting fat droplet mobility and reducing coalescence [49]. From a technological standpoint, improved lipid stabilization is important because it supports a more uniform emulsion structure and may reduce fat exudation during heat treatment.

At the same time, the increase in cooking loss at higher replacement levels indicates that emulsion stabilization alone was not sufficient to fully compensate for progressive weakening of the myofibrillar protein framework. In comminuted meat products, thermal yield depends strongly on the ability of myosin-rich protein networks to retain water and soluble components during denaturation and contraction [50]. As the proportion of muscle proteins decreases, the gel matrix becomes less effective in entrapping aqueous phases, which explains the higher cooking losses observed in the more strongly substituted samples. The slight decrease in cooking loss after holding suggests that short-term maturation improved water redistribution and protein hydration before heating, thereby contributing to better structural organization of the batter [51]. Nevertheless, the results indicate that excessive replacement shifts the balance from controlled restructuring toward reduced yield stability.

The mechanical and textural data provide further evidence of this structural transition. The progressive reduction in shear stress, cutting work, hardness, gumminess, chewiness, cohesiveness, and springiness indicates that the protein gel became less dense and less resistant to deformation as the replacement level increased. This effect can be explained by the fact that myofibrillar proteins, particularly myosin and actin, are primarily responsible for the formation of a strong three-dimensional gel network in emulsion-type sausages [52]. Plasma proteins contribute important functional properties, but their heat-induced aggregation behavior differs from that of muscle proteins and generally results in softer, less rigid structures [53]. The hydrated flax component may additionally increase matrix plasticity through viscosity enhancement and water retention. As a result, moderate replacement levels produced a softer and potentially more tender product, whereas the highest level caused excessive weakening of the thermo-induced framework, which may negatively affect slicing properties, cohesion, and consumer perception.

The nutritional results indicate that reformulation modified the quality of both protein and lipid fractions without causing marked nutritional imbalance. The gradual increase in total amino acid content and the preservation of key essential amino acids such as leucine, lysine, isoleucine, and tryptophan suggest that moderate incorporation of plasma-based composition maintained the biological value of the protein system. The increase in valine and threonine supports the conclusion that plasma proteins contributed positively to the essential amino acid profile. At the same time, the decrease in methionine + cysteine indicates that substitution of muscle proteins by plasma and plant-derived components altered the sulfur-containing amino acid fraction. This point is nutritionally relevant because sulfur amino acids may become limiting when replacement levels are excessive. Therefore, moderate inclusion appears preferable for maintaining amino acid adequacy.

A similar conclusion can be drawn from the fatty acid data. Reformulation resulted in a reduction in short- and medium-chain saturated fatty acids, whereas palmitic acid remained stable and stearic acid increased. From a nutritional perspective, this shift is not necessarily unfavorable, since stearic acid is generally considered less hypercholesterolemic than other saturated fatty acids [54]. The relative stability of MUFA, PUFA, and total ω-6 and ω-3 fractions indicates that the essential polyunsaturated lipid profile was largely preserved across formulations. Thus, the structured plasma-based composition modified the lipid fraction quantitatively but did not substantially disrupt its overall nutritional balance. These data suggest that moderate replacement can improve selected compositional characteristics while preserving nutritional functionality.

The sensory findings are particularly important because they integrate multiple quality dimensions into the final consumer response. Samples with moderate replacement levels generally remained comparable to the control in appearance, color, taste, aroma, and consistency, indicating that the structural and compositional changes at these levels were not sufficient to impair organoleptic acceptability. However, Sample 2 exhibited slightly lower sensory scores compared to Samples 1 and 3. This may be attributed to an intermediate level of structured plasma-based composition, at which the protein–water–fat matrix undergoes partial reorganization without achieving full structural stabilization. At this level, the balance between meat proteins and the introduced plasma–flaxseed system may be suboptimal, leading to minor changes in texture (slight softness or reduced cohesiveness) and flavor perception. In contrast, lower levels (Sample 1) maintain the native meat matrix, while higher moderate levels (Sample 3) promote more uniform integration of the structured system, resulting in improved structural coherence and sensory perception. By contrast, the highest replacement level resulted in significantly lower sensory scores, especially for appearance, color, taste, and consistency. This deterioration may be explained by the combined effects of pigment dilution, altered light scattering in the modified gel–emulsion system, redistribution of flavor-active compounds between aqueous and lipid phases, and reduced contribution of myofibrillar proteins to texture formation [55]. Thus, the sensory data confirm that there is a threshold beyond which functional reformulation begins to compromise product identity.

The microbiological results further support the technological feasibility of the developed formulations. All variants showed low initial microbial load, absence of major hygienic indicator organisms and pathogens, and acceptable microbial dynamics during refrigerated storage. Slightly lower total viable counts in some experimental samples may reflect changes in water distribution or availability of nutrients in the modified matrix [56]. The increase in lactic acid bacteria during storage, particularly in samples containing the structured composition, may also be associated with the fermented plasma system and could contribute to microbial stability through competitive effects and metabolite production [57]. These microbiological findings indicate that the structured plasma-based composition can be incorporated without adversely affecting product safety during refrigerated storage.

In this context, the technological relevance of the system should be considered not only from the standpoint of hygienic stability, but also in relation to its functional contribution to meat matrix formation and texture development. This result agrees with published evidence that blood-derived proteins can function as effective binders and texture formers in processed meats. In emulsion-type pork sausages, the addition of plasma proteins improved textural characteristics and increased shear force during cold storage, confirming the feasibility of plasma in meat matrices [58]. The present work differs by employing a pre-structured fermented plasma system and by targeting partial meat replacement. Nevertheless, both studies converge on the principle that plasma proteins strengthen structure when applied within an appropriate formulation window.

Comparable optimum-level behavior has also been reported for non-blood functional ingredients that improve texture by binding water and forming gel-like structures. Flaxseed flour improved binding, hardness, and shear force in restructured mutton chops, with an optimal inclusion level identified on the basis of sensory and physicochemical criteria [59]. Similarly, incorporation of flaxseed and tomato powders in sausages maintained acceptable sensory quality at low inclusion levels, while primarily delivering nutritional and storage-related advantages, including reduced residual nitrite [47]. These findings support the broader concept that functional ingredients can improve product performance, but only up to a threshold beyond which texture and sensory quality may deteriorate.

Studies on poultry sausages also show that the form of plasma and the way it is added are important. In one study, soy protein and synthetic dyes were replaced with broiler blood derivatives. The sausages remained microbiologically safe during storage. Sensory acceptance was similar to standard products when liquid plasma was used. However, freeze-dried plasma reduced acceptance [60]. This result aligns with the present observation that technological feasibility alone is insufficient; the physical form and integration of plasma proteins into the meat matrix can determine consumer response.

Taken together, the results indicate that a 25% replacement level provides a favorable balance of technological, nutritional, and sensory properties. At this level, moisture retention, emulsifying properties, nutritional composition, sensory quality, and microbiological safety remain acceptable, while the structural integrity of the sausage matrix is maintained. Therefore, the structured plasma-based composition can be considered an effective multifunctional ingredient when applied within an appropriate formulation range.

## 5. Conclusions

This study demonstrated that incorporation of a pre-structured bovine plasma–flaxseed composition modifies the functional behavior of cooked sausage systems while maintaining technological stability at moderate inclusion levels. Water-holding capacity remained statistically unchanged (*p* > 0.05), whereas fat-holding and emulsifying capacities improved (*p* < 0.05), indicating enhanced stabilization of the lipid phase. Texture analysis revealed controlled softening, with significant reductions in hardness and chewiness at higher substitution levels (*p* < 0.05). Nutritional evaluation showed a tendency toward increased total amino acid content and moderate shifts in fatty acid profile without detrimental changes in polyunsaturated fractions. Microbiological analysis confirmed product safety during refrigerated storage. A replacement level of 15–25% provided a favorable balance of technological and sensory performance under the conditions studied. However, detailed microstructural characterization of the protein–polysaccharide system was beyond the scope of this study. Future studies may address broader consumer evaluation and further characterization of structural interactions, as well as explore the application of this system in other meat products.

## 6. Patents

Diana S. Sviderskaya, Elena F. Krasnopyorova, Asem M. Shulenova, patent for invention No. 37337, ‘Composition for the production of meat and vegetable pâté.’ Bulletin No. 22, 30 May 2025.

## Figures and Tables

**Table 1:** Formulations of emulsion-type sausages.

Components	Control	Sample 1	Sample 2	Sample 3	Sample 4
Beef	75.0	60.0	55.0	50.0	45.0
Raw beef fat	15.5	15.5	15.5	15.5	15.5
Egg mélange	5.0	5.0	5.0	5.0	5.0
Structural composition based on blood plasma	–	15.0	20.0	25.0	30.0
Table salt	2.3	2.3	2.3	2.3	2.3
Sodium nitrite	0.1	0.1	0.1	0.1	0.1
Granulated sugar	0.3	0.3	0.3	0.3	0.3
Flavoring mixture	0.8	0.8	0.8	0.8	0.8
Flavoring agent	1.0	1.0	1.0	1.0	1.0
Total	100	100	100	100	100

**Table 2:** Chemical composition of sausage variants.

Indicator	Control	Sample 1	Sample 2	Sample 3	Sample 4
Water, %	66.58 ± 0.92 ^a^	67.81 ± 1.63 ^a^	68.81 ± 0.94 ^ab^	69.82 ± 0.86 ^ab^	70.95 ± 0.87 ^b^
Protein, %	17.90 ± 0.28 ^c^	17.20 ± 0.26 ^bc^	16.40 ± 0.21 ^ab^	15.70 ± 0.27 ^a^	15.20 ± 0.18 ^a^
Fat, %	12.10 ± 0.28 ^c^	11.50 ± 0.12 ^bc^	11.20 ± 0.23 ^ab^	10.90 ± 0.16 ^a^	10.20 ± 0.13 ^a^
Carbohydrates, %	1.42 ± 0.02 ^a^	1.42 ± 0.02 ^a^	1.43 ± 0.03 ^a^	1.37 ± 0.02 ^a^	1.40 ± 0.03 ^a^
Ash, %	2.00 ± 0.04 ^a^	2.07 ± 0.04 ^ab^	2.16 ± 0.04 ^b^	2.21 ± 0.06 ^b^	2.25 ± 0.04 ^b^

^a–c^ Different lowercase letters indicate statistically significant differences within the rows (*p* < 0.05).

**Table 3:** Effect of structured blood plasma-based composition on shear stress and cutting work of emulsion-type sausages.

Indicator	Control	Sample 1	Sample 2	Sample 3	Sample 4
Shear stress, Pa	2684 ± 33 ^d^	2380 ± 39 ^c^	2274 ± 47 ^bc^	2185 ± 46 ^ab^	2055 ± 35 ^a^
Cutting work, J/m^2^	210.50 ± 3.5 ^d^	194.71 ± 3.0 ^c^	188.65 ± 2.8 ^bc^	176.10 ± 2.9 ^b^	158.50 ± 2.9 ^a^

^a–d^ Different lowercase letters indicate statistically significant differences within the rows (*p* < 0.05).

**Table 4:** TPA parameters of emulsion-type sausages with different levels of structured plasma-based composition.

Indicator	Control	Sample 1	Sample 2	Sample 3	Sample 4
Hardness	0.694 ± 0.014 ^e^	0.634 ± 0.013 ^d^	0.584 ± 0.008 ^c^	0.542 ± 0.011 ^b^	0.484 ± 0.008 ^a^
Adhesiveness	−0.028 ± 0.000 ^c^	−0.027 ± 0.000 ^bc^	−0.026 ± 0.000 ^b^	−0.024 ± 0.000 ^a^	−0.023 ± 0.000 ^a^
Cohesiveness	0.459 ± 0.011 ^a^	0.452 ± 0.009 ^a^	0.447 ± 0.010 ^a^	0.438 ± 0.007 ^a^	0.429 ± 0.008 ^a^
Gumminess	0.304 ± 0.005 ^e^	0.286 ± 0.005 ^d^	0.266 ± 0.003 ^c^	0.245 ± 0.004 ^b^	0.227 ± 0.005 ^a^
Chewiness	0.248 ± 0.006 ^e^	0.214 ± 0.005 ^d^	0.196 ± 0.004 ^c^	0.172 ± 0.003 ^b^	0.149 ± 0.003 ^a^
Springiness	0.148 ± 0.003 ^cd^	0.140 ± 0.003 ^c^	0.135 ± 0.002 ^c^	0.128 ± 0.002 ^ab^	0.123 ± 0.001 ^a^

^a–e^ Different letters within a row indicate statistically significant differences (*p* < 0.05).

**Table 5:** Essential amino acid composition of emulsion-type sausages with partial replacement of beef by structured blood plasma-based composition.

Amino Acid	Control	Sample 1	Sample 2	Sample 3	Sample 4
Valine	4.12 ± 0.07 ^a^	4.20 ± 0.08 ^a^	4.24 ± 0.08 ^ab^	4.35 ± 0.05 ^b^	4.38 ± 0.08 ^b^
Isoleucine	3.15 ± 0.05 ^a^	3.20 ± 0.07 ^a^	3.22 ± 0.06 ^a^	3.20 ± 0.04 ^a^	3.20 ± 0.04 ^a^
Leucine	6.95 ± 0.12 ^a^	7.05 ± 0.17 ^a^	7.10 ± 0.13 ^a^	7.14 ± 0.12 ^a^	7.20 ± 0.09 ^a^
Lysine	6.50 ± 0.10 ^a^	6.60 ± 0.12 ^a^	6.60 ± 0.08 ^a^	6.60 ± 0.11 ^a^	6.60 ± 0.10 ^a^
Methionine + Cysteine	2.40 ± 0.04 ^c^	2.37 ± 0.02 ^bc^	2.30 ± 0.04 ^bc^	2.23 ± 0.04 ^ab^	2.14 ± 0.03 ^a^
Threonine	3.86 ± 0.07 ^a^	3.98 ± 0.05 ^ab^	4.10 ± 0.08 ^ab^	4.13 ± 0.09 ^bc^	4.20 ± 0.07 ^c^
Phenylalanine + Tyrosine	6.37 ± 0.16 ^a^	6.55 ± 0.11 ^a^	6.62 ± 0.14 ^a^	6.70 ± 0.12 ^a^	6.75 ± 0.09 ^a^
Tryptophan	0.98 ± 0.02 ^a^	0.96 ± 0.01 ^a^	0.98 ± 0.02 ^a^	0.97 ± 0.02 ^a^	0.98 ± 0.01 ^a^
Histidine	2.80 ± 0.05 ^b^	2.74 ± 0.04 ^ab^	2.70 ± 0.04 ^ab^	2.65 ± 0.05 ^a^	2.63 ± 0.05 ^a^
Total amount of amino acids	37.13 ± 0.72 ^a^	37.65 ± 0.61 ^a^	37.86 ± 0.33 ^a^	37.97 ± 0.65 ^a^	38.08 ± 0.62 ^a^

^a–c^ Different lowercase letters indicate statistically significant differences within the rows (*p* < 0.05).

**Table 6:** Fatty acid composition of emulsion-type sausages with partial replacement of beef by structured blood plasma-based composition.

Fatty Acid	Control, g/100 g	Sample 1, g/100 g	Sample 2, g/100 g	Sample 3, g/100 g	Sample 4, g/100 g
C10:0 Capric	0.028 ± 0.001 ^e^	0.026 ± 0.001 ^d^	0.024 ± 0.000 ^c^	0.018 ± 0.000 ^b^	0.012 ± 0.000 ^a^
C12:0 Lauric	0.048 ± 0.001 ^c^	0.048 ± 0.001 ^c^	0.046 ± 0.001 ^c^	0.042 ± 0.001 ^b^	0.037 ± 0.001 ^a^
C 14:0 Myristic	1.687 ± 0.032 ^b^	1.674 ± 0.021 ^b^	1.655 ± 0.040 ^b^	1.644 ± 0.025 ^b^	1.438 ± 0.019 ^a^
C 16:0 Palmitic	25.10 ± 0.45 ^a^	24.53 ± 0.40 ^a^	25.53 ± 0.40 ^a^	25.10 ± 0.52 ^a^	24.99 ± 0.25 ^a^
C 18:0 Stearic	25.10 ± 0.55 ^a^	26.99 ± 0.46 ^ab^	27.55 ± 0.56 ^bc^	28.32 ± 0.41 ^bc^	29.15 ± 0.51 ^c^
C 6:1(Cis-9) Palmitoleic	4.213 ± 0.076 ^a^	3.982 ± 0.065 ^a^	4.055 ± 0.090 ^a^	4.157 ± 0.060 ^a^	4.410 ± 0.084 ^a^
C 18:2n-6c Linoleic	7.516 ± 0.088 ^a^	7.455 ± 0.121 ^a^	7.388 ± 0.166 ^a^	7.515 ± 0.180 ^a^	7.686 ± 0.081 ^a^
C 18:3n-6γ- Linolenic	1.657 ± 0.015 ^ab^	1.704 ± 0.030 ^ab^	1.725 ± 0.028 ^b^	1.659 ± 0.029 ^ab^	1.598 ± 0.026 ^a^
C 20:4n-6 Arachidonic	0.420 ± 0.007 ^ab^	0.424 ± 0.010 ^b^	0.422 ± 0.006 ^ab^	0.421 ± 0.008 ^ab^	0.395 ± 0.006 ^a^
SFA	51.97 ± 0.98 ^a^	53.27 ± 0.72 ^ab^	54.80 ± 1.18 ^ab^	55.13 ± 0.64 ^b^	55.63 ± 0.92 ^b^
MUFA	4.21 ± 0.08 ^b^	3.98 ± 0.05 ^a^	4.06 ± 0.08 ^ab^	4.16 ± 0.07 ^ab^	4.41 ± 0.06 ^c^
PUFA	9.59 ± 0.18 ^a^	9.58 ± 0.24 ^a^	9.54 ± 0.13 ^a^	9.60 ± 0.23 ^a^	9.68 ± 0.15 ^a^
Total ω-6	7.94 ± 0.14 ^a^	7.88 ± 0.10 ^a^	7.81 ± 0.12 ^a^	7.94 ± 0.10 ^a^	8.08 ± 0.13 ^a^
Total ω-3	1.66 ± 0.03 ^ab^	1.70 ± 0.03 ^ab^	1.73 ± 0.02 ^b^	1.66 ± 0.03 ^ab^	1.60 ± 0.02 ^a^
Total fatty acids	65.77 ± 1.05 ^a^	66.83 ± 1.03 ^ab^	68.39 ± 1.22 ^ab^	68.88 ± 0.98 ^ab^	69.72 ± 1.48 ^b^

^a–e^ Different lowercase letters indicate statistically significant differences within the rows (*p* < 0.05).

## Data Availability

The original contributions presented in this study are included in the article/Supplementary Materials. Further inquiries can be directed to the corresponding author.

## Supplementary Materials

The following supporting information can be downloaded at: https://www.mdpi.com/article/10.3390/foods15081336/s1, Table S1. Microbiological profile of cooked sausage formulations during 14 days of refrigerated storage.

## References

[1] Dàvila E., Saguer E., Toldrà M., Carretero C., Parés D. (2007). Surface functional properties of blood plasma protein fractions. Eur. Food Res. Technol..

[2] Gupta A.K., Fadzlillah N.A., Sukri S.J.M., Adediran O.A., Rather M.A., Naik B., Kumar V., Bekhit A.E.D.A., Ramli M.A., Jha A.K. (2024). Slaughterhouse blood: A state-of-the-art review on transforming by-products into valuable nutritional resources and the role of circular economy. Food Biosci..

[3] Silva V.D., Silvestre M.P. (2003). Functional properties of bovine blood plasma intended for use as a functional ingredient in human food. LWT-Food Sci. Technol..

[4] Toldrá F., Reig M., Mora L. (2021). Management of meat by-and co-products for an improved meat processing sustainability. Meat Sci..

[5] Furlán L.R., Padilla A.P., Campderrós M.E. (2010). Functional and physical properties of bovine plasma proteins as a function of processing and pH, application in a food formulation. Adv. J. Food Sci. Technol..

[6] Bou R., Pinotti L., Zeugolis D., Watigny A., Álvarez C. (2025). Advancing the valorisation of meat co-products in food, pet food, and biomedicine applications. Waste Manag..

[7] Cherniak M.I. (2020). Possibility of using blood plasma of slaughtered animals in new protein products. Food Process. Ind. Abstr. J..

[8] Zhu X., Tu Y., Zhao Y., Wu N., Yao Y., Chen S., Wei T., Mao J., Hu X., Wang S. (2025). Emulsion gel-based fat replacers in meat products: Structured design, processing stability, oral lubricity/flavor perception, and digestive characteristics. Food Chem..

[9] Yessimbekov Z., Kakimov A., Caporaso N., Suychinov A., Kabdylzhar B., Shariati M.A., Baikadamova A., Domínguez R., Lorenzo J.M. (2021). Use of Meat-Bone Paste to Develop Calcium-Enriched Liver Pâté. Foods.

[10] Lynch S.A., Mullen A.M., O’Neill E.E., García C.Á (2017). Harnessing the potential of blood proteins as functional ingredients: A review of the state of the art in blood processing. Compr. Rev. Food Sci. Food Saf..

[11] Choi Y.S., Sung J.M., Jeon K.H., Choi H.W., Seo D.H., Kim C.J., Kim H.W., Hwang K.E., Kim Y.B. (2015). Quality characteristics on adding blood levels to blood sausage. Korean J. Food Cook. Sci..

[12] Silva F.A.P., Amaral D.S., Guerra I.C.D., Dalmás P.S., Arcanjo N.M.O., Bezerra T.K.A., Beltrão Filho E.M., Moreira R.T., Madruga M.S. (2013). The chemical and sensory qualities of smoked blood sausage made with the edible by-products of goat slaughter. Meat Sci..

[13] Del Hoyo P., Rendueles M., Díaz M. (2008). Effect of processing on functional properties of animal blood plasma. Meat Sci..

[14] Sydykova M., Nurymkhan G., Gaptar S., Rebezov Y., Khayrullin M., Nesterenko A., Igor G. (2019). Using of lactic-acid bacteria in the production of sausage products: Modern conditions and perspectives. Int. J. Pharm. Res..

[15] Wang D., Cheng F., Wang Y., Han J., Gao F., Tian J., Zhang K., Jin Y. (2022). The Changes Occurring in Proteins during Processing and Storage of Fermented Meat Products and Their Regulation by Lactic Acid Bacteria. Foods.

[16] Zhang Q., Song X., Sun W., Wang C., Li C., He L., Wang X., Tao H., Zeng X. (2021). Evaluation and application of different cholesterol-lowering lactic acid bacteria as potential meat starters. J. Food Prot..

[17] Carneiro K.O., Campos G.Z., Scafuro Lima J.M., Rocha R.d.S., Vaz-Velho M., Todorov S.D. (2024). The Role of Lactic Acid Bacteria in Meat Products, Not Just as Starter Cultures. Foods.

[18] Bhattacharya D., Nanda P.K., Pateiro M., Lorenzo J.M., Dhar P., Das A.K. (2022). Lactic Acid Bacteria and Bacteriocins: Novel Biotechnological Approach for Biopreservation of Meat and Meat Products. Microorganisms.

[19] Wójciak K.M., Karwowska M., Dolatowski Z.J. (2014). Use of acid whey and mustard seed to replace nitrites during cooked sausage production. Meat Sci..

[20] Jin S., Choi J. (2021). Effects of porcine blood plasma on the emulsion stability, physicochemical characteristics and textural attributes of emulsified pork batter. J. Anim. Sci. Technol..

[21] Álvarez C., Drummond L., Mullen A.M. (2018). Protein recovered from meat co-products and processing streams as pork meat replacers in Irish breakfast sausages formulations. LWT.

[22] Kryzhova Y., Slobodenyuk N., Moskalenko I. (2023). Application of modern technologies to improve the quality of sausage products. Anim. Sci. Food Technol..

[23] Abdullah, Liu L., Javed H.U., Xiao J. (2022). Engineering emulsion gels as functional colloids emphasizing food applications: A review. Front. Nutr..

[24] Igenbayev A., Ospankulova G., Amirkhanov S., Aldiyeva A., Temirova I., Amirkhanov K. (2023). Substitution of Pork Fat with Beeswax-Structured Oleogels in Semi-Smoked Sausages. Appl. Sci..

[25] Baikadamova A., Kakimov A., Yessimbekov Z., Suychinov A., Turagulov R., Orynbekov D., Zhumadilova G., Zharykbasov Y. (2024). Studying the Process of Enzyme Treatment on Beef Meat-Bone Paste Quality. Appl. Sci..

[26] Lorenc F., Jarošová M., Bedrníček J., Smetana P., Bárta J. (2024). Recent trends in food and dietary applications of flaxseed mucilage: A mini review. Int. J. Food Sci. Technol..

[27] Lee J., Song H., Seo K.H., Lee H.G., Kim H., Choi M.J. (2023). Physicochemical and sensory properties of plant-based meat patties using oil-in-water emulsion. Food Biosci..

[28] Jin S.K., Choi J.S., Kim G.D. (2021). Effect of porcine plasma hydrolysate on physicochemical, antioxidant, and antimicrobial properties of emulsion-type pork sausage during cold storage. Meat Sci..

[29] Shin D.J., Yim D.G., Kwon J.A., Kim S.S., Lee H.J., Jo C. (2022). Effect of cutting time and cooking temperature on physicochemical properties of chicken breast meat emulsion sausage with olive oil. Poult. Sci..

[30] Seema L.P., Mashau M.E., Moholisa E. (2026). Nutritional composition, shelf life, and sensory characteristics of blood sausages: A review. Cogent Food Agric..

[31] Yogesh K., Langoo B.A., Sharma S.K., Yadav D.N. (2015). Technological, physico-chemical and sensory properties of raw and cooked meat batter incorporated with various levels of cold milled flaxseed powder. J. Food Sci. Technol..

[32] Ciobanu M.-M., Manoliu D.-R., Ciobotaru M.C., Flocea E.-I., Boișteanu P.-C. (2025). Dietary Fibres in Processed Meat: A Review on Nutritional Enhancement, Technological Effects, Sensory Implications and Consumer Perception. Foods.

[33] Ren Y., Huang L., Zhang Y., Li H., Zhao D., Cao J., Liu X. (2022). Application of Emulsion Gels as Fat Substitutes in Meat Products. Foods.

[34] Shulenova A., Kassenov A., Kakimov M., Kokayeva G., Mustafayeva A., Mursalykova M., Krasnopyorova Y., Sviderskaya D., Rzayev B., Iskakov B. (2025). Effect of Lactic Acid Bacteria Concentration and Flaxseed Cake Flour on the Formation and Stability of Bovine Blood Plasma Gels. Processes.

[35] (2019). Cooked Meat Sausage Products. Specifications.

[36] (2023). Meat and Meat Products—Determination of Moisture Content—Reference Method.

[37] (2023). Meat and Meat Products—Determination of Nitrogen Content—Reference Method.

[38] (1998). Meat and Meat Products—Determination of Total Ash.

[39] (2004). Meat and Meat Products—Measurement of pH—Reference Method.

[40] Nurymkhan G., Kalibekkyzy Z., Orynbekov D., Assenova B., Kambarova A., Dautova A., Maizhanova A., Zhumanova G., Atambayeva Z., Okuskhanova E. (2025). The Effect of a Multi-Component Plant Supplement on the Nutritional Value of Meat Patties. Processes.

[41] (2024). Microbiology of the Food Chain—General Requirements and Guidance for Microbiological Examinations..

[42] (2017). Meat and Meat Products. General Conditions of Organoleptical Assessment..

[43] Lorenc F., Jarošová M., Bedrníček J., Smetana P., Bárta J. (2022). Structural Characterization and Functional Properties of Flaxseed Hydrocolloids and Their Application. Foods.

[44] Zhao J., Yao X., Zhang H., Kong B., Sun F., Liu Q., Cao C. (2025). Effects of porcine plasma protein-xanthan gum based oleogels constructed by foam-templated approach as fat substitutes on the quality characteristics and flavour attributes of low-fat frankfurters. Meat Sci..

[45] Park G., Jin S., Choi J. (2022). Effects of physicochemical characteristics and storage stability of porcine albumin protein hydrolysates in pork sausage. Curr. Res. Nutr. Food Sci. J..

[46] Yu C., Chen L., Xu M., Ouyang K., Chen H., Lin S., Wang W. (2024). The effect of pH and heating on the aggregation behavior and gel properties of beef myosin. LWT.

[47] Ghafouri-Oskuei H., Javadi A., Asl M.R.S., Azadmard-Damirchi S., Armin M. (2020). Quality properties of sausage incorporated with flaxseed and tomato powders. Meat Sci..

[48] Tan S., Du M., Yuan G., Rong L., Li R., Li G. (2023). Evolution of the structure of meat protein particles at the oil–water interface facilitates the ultra-long storage stability of high internal pickering emulsion. Food Hydrocoll..

[49] Villacís-Chiriboga J., Sharifi E., Elíasdóttir H.G., Huang Z., Jafarzadeh S., Abdollahi M. (2025). Hybrid plant-based meat alternatives structured via co-extrusion: A review. Trends Food Sci. Technol..

[50] Han Z., Zhang J., Zheng J., Li X., Shao J. (2019). The study of protein conformation and hydration characteristics of meat batters at various phase transition temperatures combined with Low-field nuclear magnetic resonance and Fourier transform infrared spectroscopy. Food Chem..

[51] Nie Y., Xiong Y.L., Jiang J. (2025). Texture, microstructure, and in vitro digestion of hybrid meat gel-type sausages formulated with functionalized pea protein. Food Hydrocoll..

[52] Herrero A.M., De la Hoz L., Ordóñez J.A., Herranz B., De Ávila M.R., Cambero M.I. (2008). Tensile properties of cooked meat sausages and their correlation with texture profile analysis (TPA) parameters and physico-chemical characteristics. Meat Sci..

[53] Zou Y., Wang L., Wang X., Lan Y., Ma J., Yang J., Xu W., Shen Q., Wang D. (2024). Effect of ultrasound combined with plasma protein treatment on the structure, physicochemical and rheological properties of myofibrillar protein. Ultrason. Sonochem..

[54] Shen X., Miao S., Zhang Y., Guo X., Li W., Mao X., Zhang Q. (2025). Stearic acid metabolism in human health and disease. Clin. Nutr..

[55] Idyryshev B., Nurgazezova A., Assirzhanova Z., Utegenova A., Amirkhanov S., Jumazhanova M., Baikadamova A., Dautova A., Spanova A., Serikova A. (2025). Nutritional and Technological Benefits of Pine Nut Oil Emulsion Gel in Processed Meat Products. Foods.

[56] Rabby M.G., Islam M.N., Hasan M.S., Hossain M.N., Parvin R., Zahid M.A. (2025). Effects of Sesame and Cinnamon extract on physicochemical characteristics, oxidative stability, and antimicrobial activity in fresh poultry meatballs at refrigerated storage. Poult. Sci..

[57] Stegmayer M.A., Sirini N.E., Ruiz M.J., Soto L.P., Zbrun M.V., Lorenzo J.M., Signorini M.L., Frizzo L.S. (2023). Effects of lactic acid bacteria and coagulase-negative staphylococci on dry-fermented sausage quality and safety: Systematic review and meta-analysis. Meat Sci..

[58] Kim S., Jin S., Choi J. (2017). Effects of the addition of blood plasma proteins on physico-chemical properties of emulsion-type pork sausage during cold storage. J. Sci. Food Agric..

[59] Sharma H., Sharma B.D., Mendiratta S.K., Talukder S., Ramasamy G. (2014). Efficacy of flaxseed flour as bind enhancing agent on the quality of extended restructured mutton chops. Asian-Australas. J. Anim. Sci..

[60] Oro C.E.D., Rigo D., Gaio I., Valduga E., Paliga M., Silva M.F., Vedovatto F., Zabot G.L., Tres M.V. (2018). Formulation of chicken sausages with broiler blood proteins and dye. J. Food Sci. Technol..

