# Supplementary material for: Influence of Structured Plasma-Based Composition on Functional, Textural and Sensory Characteristics of Emulsion-Type Sausages
Source: Foods. 2026 Apr 12;15(8):1336. doi: 10.3390/foods15081336 (PMC13114544; doi:10.3390/foods15081336)
Supplement: Supplementary file 1 [file foods-15-01336-s001.zip › foods-4208621-supplementary.pdf]

Table S1. Microbiological profile of cooked sausage formulations during 14 days of refrigerated storage.

Indicator	Control	Sample 1	Sample 2	Sample 3	Sample 4
	0 day (before storage)				
Total mesophilic aerobic and facultative anaerobic microorganisms (TVC)	0.80±0.01^{a}	0.90±0.02^{b}	0.95±0.02^{b}	1.03±0.03^{c}	1.23±0.03^{d}
Total number of mesophilic lactic acid bacteria (LAB)	0.95±0.02^{a}	1.15±0.02^{b}	1.22±0.03^{b}	1.25±0.02^{bc}	1.75±0.02^{d}
Enterobacteriaceae	<1.0	<1.0	<1.0	<1.0	<1.0
Coliforms	ND	ND	ND	ND	ND
Escherichia coli	ND	ND	ND	ND	ND
Coagulase-positive staphylococci (including *Staphylococcus aureus*)	ND	ND	ND	ND	ND
Yeast and mold	<1.0	<1.0	<1.0	<1.0	<1.0
Salmonella spp.	ND	ND	ND	ND	ND
Listeria monocytogenes/Listeria spp.	ND	ND	ND	ND	ND
	7days				
Total mesophilic aerobic and facultative anaerobic microorganisms (TVC)	2.90±0.04^{b}	2.85±0.04^{b}	2.78±0.04^{ab}	2.70±0.04^{a}	2.65±0.03^{a}
Total number of mesophilic lactic acid bacteria (LAB)	2.05±0.04^{a}	2.40±0.04^{b}	2.60±0.05^{c}	2.75±0.05^{cd}	2.90±0.06^{d}
Enterobacteriaceae	<1.0	<1.0	<1.0	<1.0	<1.0
Coliforms	ND	ND	ND	ND	ND
Escherichia coli	ND	ND	ND	ND	ND
Coagulase-positive staphylococci (including *Staphylococcus aureus*)	ND	ND	ND	ND	ND
Yeast and mold	<1.0	<1.0	<1.0	<1.0	<1.0
Salmonella spp.	ND	ND	ND	ND	ND
Listeria monocytogenes/Listeria spp.	ND	ND	ND	ND	ND
	14days				
Total mesophilic aerobic and facultative anaerobic microorganisms (TVC)	3.95±0.06^{c}	3.70±0.10^{b}	3.55±0.07^{b}	3.40±0.10^{a}	3.25±0.09^{a}
Total number of mesophilic lactic acid bacteria (LAB)	3.10±0.06^{a}	3.50±0.08^{b}	3.70±0.07^{bc}	3.85±0.10^{cd}	4.00±0.12^{d}
Enterobacteriaceae	<1.0	<1.0	<1.0	<1.0	<1.0
Coliforms	ND	ND	ND	ND	ND
Escherichia coli	ND	ND	ND	ND	ND
Coagulase-positive staphylococci (including *Staphylococcus aureus*)	ND	ND	ND	ND	ND
Yeast and mold	<1.0	<1.0	<1.0	<1.0	<1.0
Salmonella spp.	ND	ND	ND	ND	ND
Listeria monocytogenes/Listeria spp.	ND	ND	ND	ND	ND

$^{a-e}$Different lowercase letters indicate statistically significant differences within the rows ($p<0.05$)

ND – not detected
